# DNA methylation mediates neural processing after odor learning in the honeybee

**DOI:** 10.1038/srep43635

**Published:** 2017-02-27

**Authors:** Stephanie D. Biergans, Charles Claudianos, Judith Reinhard, C. Giovanni Galizia

**Affiliations:** 1 Queensland Brain Institute, The University of Queensland, Australia; 2 Neurobiologie, Universität Konstanz, Germany; 3 Monash Institute of Cognitive and Clinical Neuroscience, Faculty of Medicine, Nursing Health and Sciences, Monash University, Australia

## Abstract

DNA methyltransferases (Dnmts) - epigenetic writers catalyzing the transfer of methyl-groups to cytosine (DNA methylation) – regulate different aspects of memory formation in many animal species. In honeybees, Dnmt activity is required to adjust the specificity of olfactory reward memories and bees’ relearning capability. The physiological relevance of Dnmt-mediated DNA methylation in neural networks, however, remains unknown. Here, we investigated how Dnmt activity impacts neuroplasticity in the bees’ primary olfactory center, the antennal lobe (AL) an equivalent of the vertebrate olfactory bulb. The AL is crucial for odor discrimination, an indispensable process in forming specific odor memories. Using pharmacological inhibition, we demonstrate that Dnmt activity influences neural network properties during memory formation *in vivo*. We show that Dnmt activity promotes fast odor pattern separation in trained bees. Furthermore, Dnmt activity during memory formation increases both the number of responding glomeruli and the response magnitude to a novel odor. These data suggest that Dnmt activity is necessary for a form of homoeostatic network control which might involve inhibitory interneurons in the AL network.

The morphology and physiology of the neural network underlying olfactory processing and memory formation has been studied in great detail in honey bees^1^. In the primary olfactory center (antennal lobe, AL), odor information is coded in a spatiotemporal pattern of glomerular activity, which suggests a crucial role of the AL in odor identity processing. Indeed, the representations of individual odors are more distinct after processing in the AL^2^. AL processing is accomplished primarily by a network of inhibitory local interneurons (LNs), as shown by modelling^3^ and by using GABA receptor blockers^4^,^5^,^6^. Odor response patterns separate fast and reach their maximum discriminability about 150 ms after odor onset in the AL output neurons (projection neurons, PNs)^7^,^8^. Behavioral and physiological studies suggest that bees indeed use this early information for odor discrimination^9^,^10^,^11^,^12^. The AL is also involved in olfactory memory formation^13^,^14^,^15^,^16^,^17^.

Even though olfactory memory formation has been extensively studied at both the physiological and behavioral level, many molecular aspects are poorly understood. Particularly, the dynamics of transcriptional regulation that impact neural processing and underpin memory formation remain largely unknown. Recent studies have shown that DNA methylation catalyzed by DNA methyltransferases (Dnmts) regulates stimulus-specific long-term memory (LTM) formation^18^,^19^,^20^ and relearning in bees^20^,^21^. *Dnmt1b, Dnmt3* and the Ten-eleven translocation methylcytosine dioxygenase (*Tet*), which catalyzes active demethylation, were found upregulated in a specific temporal order following olfactory reward conditioning^19^. This finding highlights a dynamic relationship between methylation and demethylation during memory formation. We proposed earlier that Dnmts may normalize transcription levels of genes activated during memory formation, in order to avoid excess neural activity and connectivity^19^. Similarly, Tet-mediated active demethylation is involved in synaptic scaling, a mechanism of homeostatic plasticity, a slow cell- and/or neural network-wide form of neural plasticity^22^,^23^.

To understand how Dnmt activity mediates learning-related plasticity in neural networks, we investigated odor responses in the AL output neurons (PNs) with and without Dnmt activity during memory formation, *in vivo*. We inhibited Dnmt activity using the non-specific Dnmt inhibitor RG108^24^,^25^,^26^, which has been repeatedly used in behavioral and neural plasticity studies^19^,^20^,^23^,^27^,^28^,^29^,^30^. Treatment with RG108 following olfactory reward learning reduces global DNA methylation in the honeybee brain and affects the expression of memory-associated genes^19^.

Dnmt inhibition impaired odor response pattern separation between a trained and a new odor. Furthermore, the overall number of glomeruli responsive to a new odor and their response strength was reduced after Dnmt inhibition. Interestingly, inhibiting Dnmts did not change the response to the learned odor. These results suggest that Dnmts are involved in regulating plasticity in the inhibitory neural network of the AL during memory formation.

## Results

### Dnmt inhibition impairs stimulus-specific memory formation in bees

**Figure 1:**
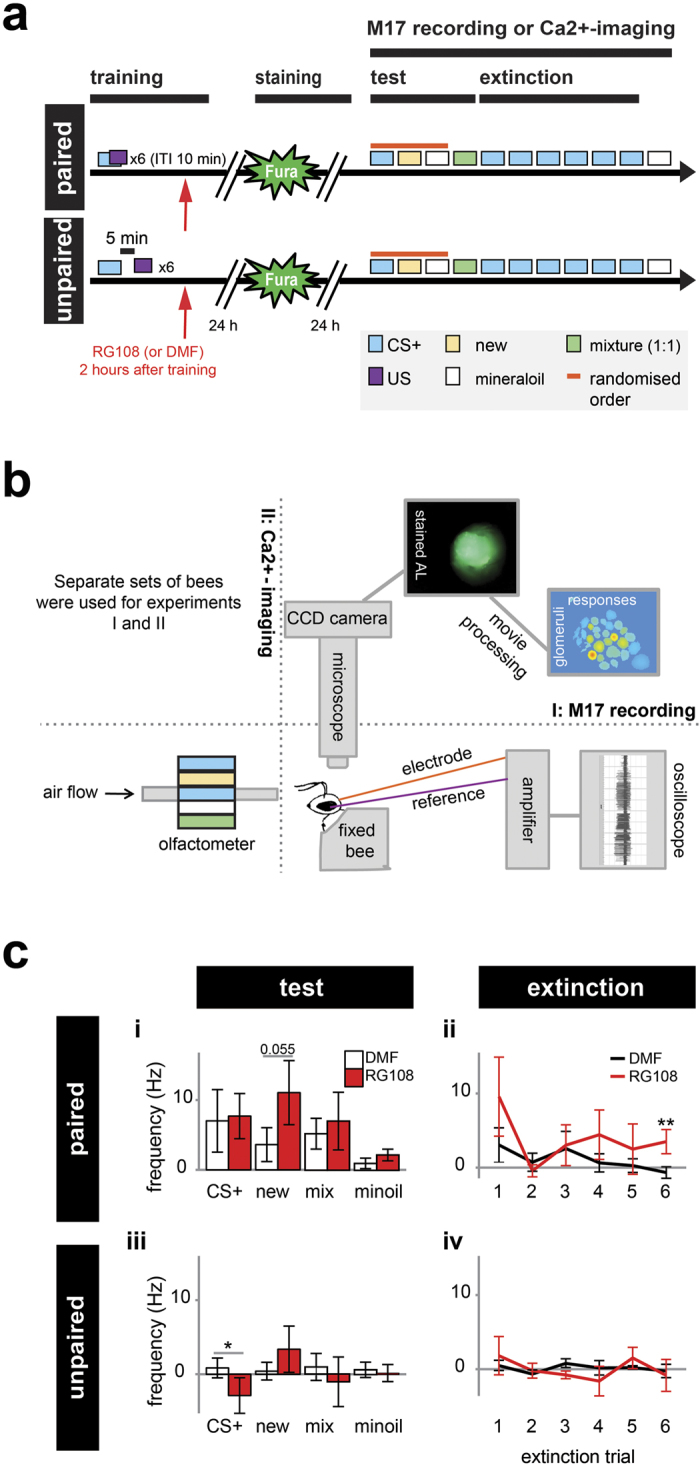
RG108 treatment impairs stimulus-specific memory and extinction in bees. (**a**) 2 hours after either paired or unpaired training bees were treated with the Dnmt inhibitor RG108 or the solvent DMF (red arrow). 1 day after the training PNs were stained with a Ca^2+^-sensitive dye (Fura). 1 day following the staining, bees were exposed to odors (**b**) while either their M17 or AL activity was recorded. First, bees were exposed to the trained odor (CS+), a new odor and the odor solvent mineraloil (min) in randomized order followed by the binary mixture of CS+ and new. After that bees received extinction training (6× CS+). (**b**) Bees’ AL was imaged using a fluorescence microscope with an attached CCD camera. In a separate group of bees the PER muscle (M17) activity was recorded as a behavioral control of Dnmt inhibition efficiency. (**c**) The M17 spike frequency (mean +/− SEM) is shown 2 days after conditioning. RG108 treated bees responded more to the new odor compared to solvent treated bees. Extinction learning was impaired in the last extinction trial in the paired group. Number of bees: paired n(RG108) = 19, n(DMF) = 26; unpaired n(RG108) = 15, n(DMF) = 13; *is p < 0.05, **is p < 0.01.

Behavioral studies in bees show that Dnmts are involved in stimulus-specific LTM formation^18^,^19^,^20^. When Dnmts are active following olfactory reward conditioning, stimulus-specific memory increases and bees generalize less to a novel odor. The neural network properties regulated by Dnmts during LTM formation remain unknown, however. We hypothesized that Dnmts mediate learning-related plasticity in the honeybee primary olfactory center (antennal lobe, AL) and thus strengthen stimulus-specific memory formation in this neuropil. To test this hypothesis, we combined the use of a non-specific Dnmt inhibitor, RG108^24^,^25^,^26^, with *in vivo* Ca^2+^-imaging of the AL output neurons (projection neurons, PNs). RG108 treatment reduces DNA methylation levels in the bee brain and affects memory-associated gene expression^19^. As in the former study, bees were treated with the inhibitor or the solvent (DMF) 2 hours after olfactory reward conditioning (Fig. 1a). We tested two behavioral groups, paired and unpaired: in paired training the conditioned (CS) and unconditioned stimulus (US) overlapped 2 s, and trials were separated by a 10 minute interval. In unpaired training (i.e. stimuli control), there was a 5 minute gap between CS and US.

Bees were trained on day 1, stained with the calcium sensitive dye FURA on day 2, and tested on day 3 (Fig. 1a). We used electrophysiological recordings from the bees proboscis muscle (M17) 2 days after conditioning in order to assess memory retention^31^ and to confirm the effect of Dnmt inhibition (Fig. 1b). We followed the same protocol as in the Ca^2+^-imaging experiment to confirm that the experimental treatment (e.g. staining) does not affect the previously described effect of Dnmt inhibition on memory formation. Solvent treated bees responded strongly to the trained odor (mean firing rate during 4 s stimulus: 7 Hz ± 4.5 Hz), but weakly to the empty stimulus mineral oil (1 Hz, ± 0.8 Hz), showing that the bees had learned to respond to the trained odor (Fig. 1c(i), one-tailed Wilcoxon signed rank test: p = 0.065, effect size (d) = 0.435). There was no effect of the Dnmt inhibitor on the CS+ response (Fig. 1c(i), Mann-Whitney U test: p = 0.99), confirming previous results^18^. As expected, there was no learning in the unpaired group for either treatment (Fig. 1c(iii), one-tailed Wilcoxon signed rank test: DMF: p = 0.345, RG108: p = 0.979). Additionally, we tested the bees’ responses to a new odor in order to test for stimulus-specific memory. RG108-treated bees generalized more to a new odor compared to solvent treated bees (Fig. 1c(i), Mann-Whitney U test: p = 0.055, d = 0.464), confirming previous data^18^,^19^,^20^. As Dnmts were also found to promote extinction learning^21^, we exposed bees to the CS+ 6 times following the memory test (Fig. 1a). Inhibiting Dnmts with RG108 led to less extinction learning with RG108 and DMF treated bees differing significantly in the 6^th^ extinction trial (Fig. 1c(ii), Mann-Whitney U test: p = 0.006, d = 0.243). Taken together, measurements of M17 responses in our preparation confirmed previously published data, and showed that the experimental treatments used here (in particular, staining with FURA, and keeping the bees in the recording chamber for three days) did not affect the bees’ capacity to learn, and did not modify the effect of Dnmts on memory formation and extinction.

### Dnmt inhibition impairs fast odor identity processing following olfactory reward conditioning

**Figure 2:**
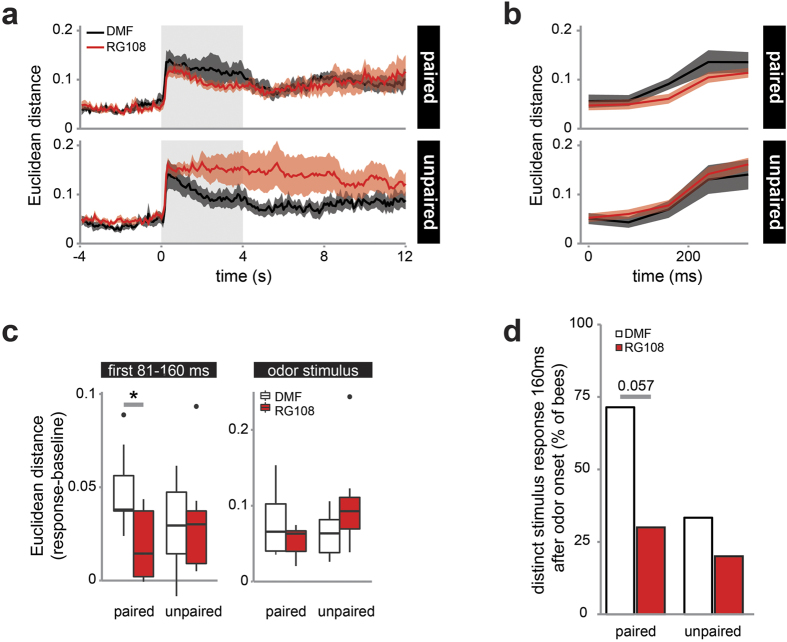
Dnmt inhibition impairs fast odor response pattern separation. (**a**) Odor stimuli (shaded area) elicited significant responses in all groups (Euclidean distance as dissimilarity measure, mean +/− SEM). (**b**) Odor-specific patterns established at a slower pace when Dnmts were inhibited: 81–160 ms after odor onset dissimilarity was smaller in the paired/RG108 group. (**c**) The Euclidian distance was significantly different between treatments in the paired, but not the unpaired group. There was no difference when considering the entire odor stimulus. (**d**) More bees established distinct odor response patterns (Euclidean distance >3× SD of baseline) within the first 160 ms after odor onset in paired control bees, compared to paired RG108 treated bees. Number of bees: paired: n(RG108) = 10, n(DMF) = 7; unpaired: n(RG108) = 10, n(DMF) = 6; *is p < 0.05.

We recorded odor responses in PNs 2 days after training (Fig. 1a,b). Since Dnmts have been implicated with odor generalization after learning, we first analyzed how similar the responses to two different odors were. We calculated the Euclidean distance (i.e. dissimilarity measure) between the odor response patterns to the CS+ and a new odor 2 days after olfactory reward conditioning (Fig. 2a). Background dissimilarity (noise) was in the range of 0.05. Upon odor stimulation, the dissimilarity increased to above 0.1, and decreased slowly thereafter. In the paired group, Dnmt inhibition led to less distinct odor patterns upon stimulus presentation (Fig. 2a). This effect was most prominent in the initial odor response (Fig. 2b): following Dnmt inhibition the Euclidean distance decreased within the first **81–160 ms** (Fig. 2c, t-test: paired: p = 0.019, d = 1.408, Mann-Whitney U test: unpaired: p = 0.875). When averaged across the whole odor period, however, there was no significant difference between treatments (Fig. 2c, Mann-Whitney U test: group: paired: p = 0.364; unpaired: p = 0.073).

To confirm that RG108 treatment following learning reduces odor pattern separation within the first 160 ms after odor onset, we calculated the proportion of bees showing distinct odor patterns in that time period (Euclidean distance >3× SD of baseline). More than 70% of trained control bees showed distinct odor patterns compared to only 30% of bees following RG108 treatment (Fig. 2d, one-sided Mann-Whitney U test: p = 0.057). We conclude that Dnmt activity modifies the antennal lobe neural network during memory formation in a way that could allow for faster odor pattern discrimination.

### Dnmt inhibition during memory formation decreases the number of glomeruli responding to a new odor

**Figure 3:**
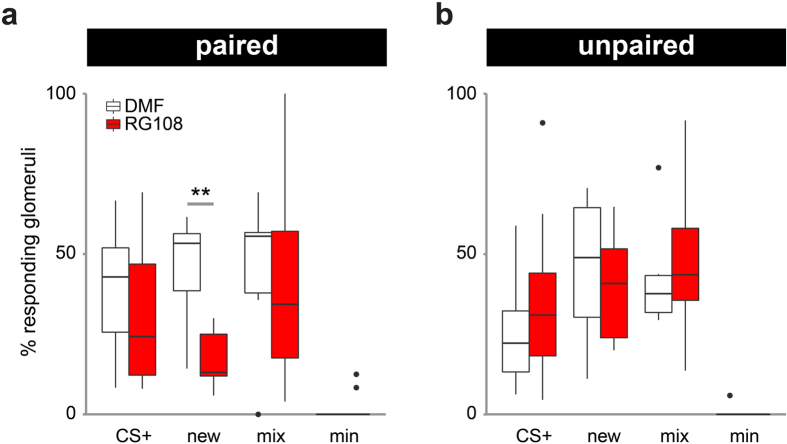
Dnmt inhibition during memory formation decreases the number of glomeruli responding to a new odor. For each bee the % of glomeruli responding to each odor stimulus is plotted. Responding glomeruli were defined using the same criterion as described before^32^. (**a**) Less glomeruli responded to the new odor after RG108 treatment in the paired group, (**b**) but not in the unpaired group. Number of bees: paired: n(RG108) = 10, n(DMF) = 7; unpaired: n(RG108) = 10, n(DMF) = 6; **is p < 0.01.

Odor learning can change the odor response strength in olfactory glomeruli depending on their activity during training^32^. Therefore, we quantified the percentage of activated glomeruli in the memory test for each stimulus as described previously^32^. More glomeruli responded to the new odor after learning than to the CS+ (Fig. 3a). However, this effect was reversed when Dnmts were inhibited during memory formation: fewer glomeruli responded to the new odor, as compared to solvent treated bees (Fig. 3a, t-test: p = 0.003, d = 2.248). The number of odor-activated glomeruli in response to the new odor did not change when Dnmts were inhibited in the unpaired group (Fig. 3b, t-test: p = 0.566). This analysis shows that Dnmts are involved in recruiting additional glomeruli into the responses to new odors after learning.

### Dnmt inhibition during memory formation leads to weaker responses of glomeruli to a new odor

**Figure 4:**
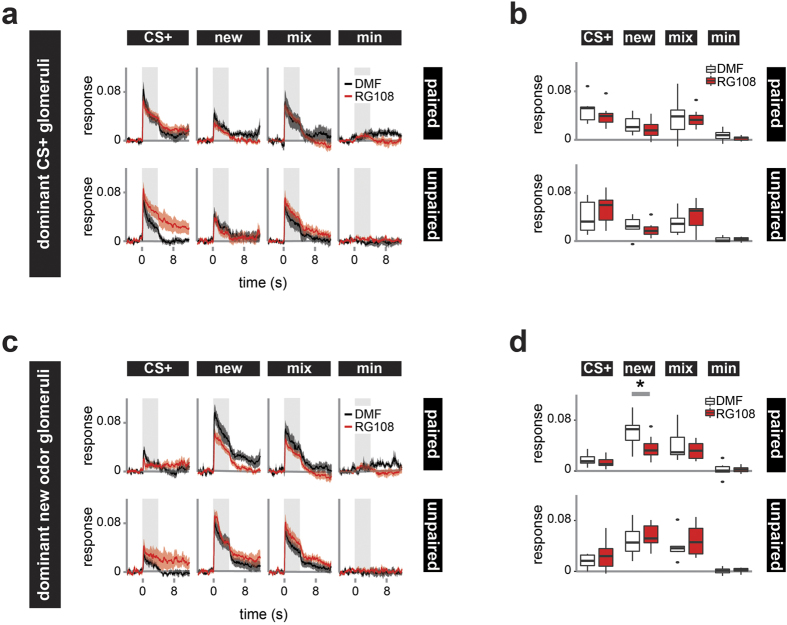
Dnmt inhibition during memory formation decreases the response strength of glomeruli strongly responding to the new odor. Response strength of glomeruli was assessed by analyzing those two glomeruli responding most to the (**a**,**b**) CS+ and (**c**,**d**) the new odor respectively (dominant glomeruli). (**a**) For the dominant CS+ glomeruli, the average response over time and (**b**) pooled across the odor peak is shown. The responses did not significantly differ between RG108 (red) and DMF (black) treated bees in the paired or unpaired group. (**c**,**d**) The responses of dominant new odor glomeruli differed between treatments in the paired group. (**d**) The response was weaker in RG108 treated bees when stimulated with the new odor. This, however, was not the case in unpaired bees. (**a**,**c**) The mean (+/−SEM) is shown. The shaded area indicates the odor stimulus. Number of bees: paired n(RG108) = 10, n(DMF) = 7; unpaired n(RG108) = 10, n(DMF) = 6; *is p < 0.05.

Next we wanted to know whether Dnmt activity also affects the odor responses of glomeruli strongly responding to the test odors. We focused on the two most dominant glomeruli for responses to the CS+ and the new odor, respectively (Fig. 4). As expected, the dominant CS+ glomeruli showed weaker responses to the new odor, and intermediate responses to the mixture of the two odors (Fig. 4a). There, however, was no significant difference for either paired or unpaired bees between treatments (Fig. 4b). Glomeruli most responsive to the new odor, on the other hand, showed a decreased response to this odor when Dnmts had been inhibited during memory formation (Fig. 4c). This difference was significant during the odor peak in the paired group, while there was no difference in the unpaired group (Fig. 4d, t-test: paired: p = 0.038, d = 1.258, unpaired: p = 0.628).

### Dnmt inhibition during memory formation does not affect odor responses in the AL during extinction learning

Extinction learning occurs when a learned stimulus is presented repeatedly without reinforcement. Extinction can be influenced by Dnmts^21^. We exposed bees to the CS+ six times following the memory test (Fig. 1a). We calculated how the representation of the CS+ changed during these six presentations by calculating the Euclidean distance relative to the first presentation (0 = stable odor response). Odor responses changed slightly with accumulating extinction trials, but treatment groups did not differ.

**Figure 5:**
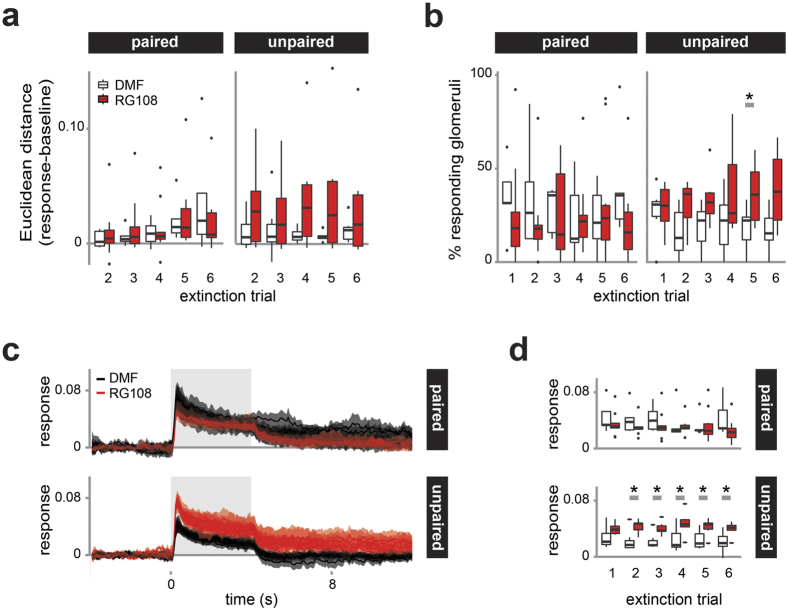
Dnmt inhibition does not affect odor responses during extinction learning in the AL. Bees were exposed to the CS+ six times, which causes extinction learning and a reduced PER response. (**a**) the Euclidean distance in relation to the first extinction trial did not differed between RG108 (red) and solvent (white) treated bees. (**b**) The number of active glomeruli changed in the unpaired group during the 5^th^ extinction trial. (**c**) The averaged response (+/−SEM) of the dominant CS+ glomeruli is shown over time for all six trials. In paired bees the response was similar in RG108 (red) and DMF treated bees (black). In unpaired bees, however, the response during and after the odor stimulus is increased after Dnmt inhibition. (**d**) During the odor peak, response strength increased following Dnmt inhibition for five out of six extinction trials in the unpaired group. Number of bees: paired n(RG108) = 10, n(DMF) = 5; unpaired n(RG108) = 6, n(DMF) = 7; *is p < 0.05.

The number of responding glomeruli was significantly higher following RG108 treatment in the unpaired group in the 5^th^ extinction trial (Fig. 5b, t-test: p = 0.050, d = 1.312). Additionally, the two dominant glomeruli increased their response strength to the repeated stimulus in the unpaired/RG108 group (Fig. 5c). This effect was significant during the odor peak in the 2^nd^–6^th^ extinction trial (Fig. 5d; Mann-Whitney U test: 2^nd^ trial: p = 0.022, d = 1.673; 3^rd^: p = 0.014, d = 1.750; 4^th^: p = 0.040, d = 1.314; 5^th^: p = 0.030, d = 1.382; 6^th^: p = 0.023, d = 1.439). Our data indicate that glomeruli responses remained largely stable over repeated odor stimulations. At least in the unpaired group (no learning, but pre-exposure) this stability necessitated a Dnmt-dependent mechanism.

## Discussion

Here we investigated whether and how Dnmts mediate plasticity in the honeybee AL after olfactory reward conditioning and during extinction. Using Ca^2+^-imaging of odor evoked PN activity in the AL we show that Dnmt inhibition during memory formation impairs the number and response strength of glomeruli responding to a new odor. Additionally, the dynamics of odor pattern separation between the CS+ and a new odor changed depending on Dnmt activity during memory formation. Furthermore, AL responses during extinction learning were not affected by Dnmt inhibition. After stimulation alone, however, Dnmt inhibition impaired a stable response of glomeruli with repeated presentations of the pre-exposed odor.

### Dnmt activity promotes stimulus-specific memory formation by facilitating fast odor pattern separation

The findings described here can be directly connected to what we know about the function of DNA methyltransferase dependent DNA methylation in memory formation in honeybees from behavioral studies. Dnmt activity promotes stimulus-specific LTM formation after multiple-trial olfactory reward conditioning^18^,^19^,^20^. We could show here that Dnmt inhibition impairs fast odor response pattern separation between a learned and a new odor. Odor discrimination in the AL is fast and maximum pattern separation is reached around 150 ms after odor onset in PNs^7^,^8^. Bees respond behaviorally to trained odors within 430–470 ms^9^,^10^. Furthermore, bees can successfully discriminate odors, even if they smell them for just 200 ms^9^. This suggests that bees use information about odor pattern similarity which is generated during the first few hundred milliseconds, in order to decide whether to respond to an odor or not. An associative change in the temporal dynamics of odor pattern separation - mediated by DNA methyltransferases - would have a strong impact on generalization between odors and thus stimulus-specific memory.

### Dnmt activity might affect memory-related plasticity in local inhibitory neurons of the AL

Interestingly, inhibition of Dnmt activity during memory formation did not globally affect response strength; it rather specifically decreased the number and strength of glomeruli not strongly active during the training. The specificity of the effect suggests that the bees’ health did not bias the results, although the mortality of bees was high. Furthermore, mortality was similar in the different treatment groups (Supplemental Tables 1 and 2).

Our results allow speculation that Dnmts might regulate the strength of inhibitory connections from CS+ glomeruli to those weakly active or inactive during training. The majority of inhibitory LNs in the AL are heterogeneous, branching strongly in one glomerulus and weakly in few others^33^,^34^. Indeed, the glomeruli most active in response to the two odors used here have inhibitory connections onto each other^2^,^35^. Additionally, it has been suggested earlier that heterogeneous LNs are plastic following olfactory reward learning^3^,^36^ and that they play a crucial role in odor discrimination^4^,^5^. Alternatively, or additionally, synaptic plasticity might occur in a glomerular subpopulation of output synapses in homogeneous LNs, yielding a spatially complex functional pattern.

Intriguingly, Dnmt inhibition did not change the response to the CS+. This result is consistent with behavioral data showing that Dnmt activity is not required for forming the association between the CS and US, but rather affects memory specificity (Fig. 1c, refs 18, 19, 20). One possible explanation for this phenomenon is that Dnmts might not be active in the neural network and/or at the time-point relevant for CS+ memory formation.

Indeed, there is evidence for increased *Dnmt3* expression 5 hours after olfactory learning, but not earlier^19^. Dnmt activity is associated with decreased expression of memory-associated synaptic genes (e.g. *actin* and *neurexin I*)^19^, after initial expression waves during the first hours after olfactory reward learning^37^. This process might be important for restricting synaptic plasticity in the LN network after olfactory reward training, creating a temporal window for learning induced synaptic changes, followed by a temporal window for homeostatic regulation.

If Dnmts predominantly regulate plasticity in the AL LN network, then the contribution of Dnmt activity to olfactory memory specificity should depend on the degree of inhibitory connections between the glomeruli responding to the CS+ and a new odor. Therefore an important next step is to test the relationship between associative plasticity in the inhibitory local AL network and Dnmt activity, ideally by recording directly from LNs.

### Dnmt activity might serve distinct regulatory functions following learning and odor exposure

Here we investigated the role of DNA methyltransferases in both animals which formed memories and those which were stimulated with odor and sugar repeatedly, but did not form memories. The differences we found between these two groups highlight two interesting aspects of how Dnmts might regulate transcription-dependent plasticity in the AL: (1) part of the regulation is memory-dependent. This is supported by evidence that some memory-associated genes show learning-dependent changes in their methylation pattern^19^. (2) Dnmt-dependent plasticity after learning and stimulation had different characteristics, as in one case the immediate response to a new odor changed, and in the other the repeated response to the pre-exposed odor. This suggests that Dnmts may have two distinct roles in this context: first; to restrict gene expression levels during memory formation^19^ and second; to regulate re-expression of genes^38^. Additionally, different genes could be targeted by Dnmts under different circumstances: in some genes Dnmt-mediated DNA methylation changes occur exclusively in response to learning, and in others in response to both learning and stimulation or to stimulation only^19^.

### Dnmt activity might contribute to homeostatic plasticity acting on the level of whole cells and neural networks hours and days after training

Different types of plasticity can occur following neural activity, including immediate Hebbian and protracted homeostatic plasticity^39^. Homoeostatic plasticity globally counteracts activity-induced local changes in order to normalize overall activity levels and prevent extrema^39^,^40^,^41^. Homoeostatic plasticity is induced by and utilizes mechanisms (e.g. intracellular Ca^2+^ levels) which overlap those utilized for long-term potentiation (LTP, i.e. the cellular equivalent of LTM)^39^. The important distinction, however, lies in the time-scale they are acting on, as homeostatic plasticity operates within hours and days, instead of seconds^39^,^41^. Furthermore, in contrast to local synapse-specific changes, homeostatic plasticity acts globally on the whole cell or neural network. Neural network models suggest that homoeostatic plasticity is important for counteracting accelerating activity by preventing positive feedback loops^42^,^43^. Recent evidence suggests that DNA methylation levels can control synaptic scaling, a mechanism of homeostatic plasticity, *in vitro* in mammals^22^,^23^. In bees, Dnmt-dependent DNA methylation might also regulate homeostatic plasticity: *Dnmts* are upreglated on a time-scale corresponding to that of homeostatic rather than Hebbian plasticity following olfactory reward learning^19^. Furthermore, Dnmts are involved in the downregulation of a subset of memory-associated genes during olfactory memory formation^19^. We earlier proposed that Dnmts may act by normalizing the expression patterns of target genes following an initial upregulation after olfactory learning^19^. At the molecular level, such a process could contribute to homoeostatic plasticity aiming at reducing overall cell activity, excitability and synaptic growth back to baseline levels by normalizing transcription levels. Furthermore, our results suggest that plasticity in inhibitory LNs might be mediated by Dnmts. Inhibitory neurons perform a crucial function for network homeostasis, as they regulate overall activity in a network by adjusting inhibition^41^. Although a function of Dnmts in homeostatic plasticity is still speculative, our observations provide a credible starting point to address the role of epigenetic transcriptional regulators in governing the dynamics of neural networks during memory formation.

## Material and Methods

### Olfactory training and treatment

Honey bees (*Apis mellifera*) were trained using appetitive olfactory classical conditioning as described before^18^,^19^. In short, bees received six trials of odor (conditioned stimulus, CS) and sugar (unconditioned stimulus, US) pairings. In the paired group of bees the CS and US overlapped for 2 s, which causes stable long-term memory formation. In the unpaired group bees received the CS and US with a 5 minute gap between stimuli, which does not cause LTM or conditioned inhibition^44^. In the paired group bees responded to the CS+ on average in 4.5 out of 6 training trials and in the unpaired group in 0.15 out of 6 (Table 1). Both groups were trained in parallel, to avoid the influence of seasonal and day-to-day variability. In both groups the CS lasted 4 s and the US (1 M sugar water) 3 s. The US was delivered by touching the bee’s antennae with a metal pin coated with sugar water, eliciting a proboscis extension response (PER) and allowing the bee to drink. Sugar water was prepared in 1 M solution (Sucrose in Water) and frozen until usage. The CS was either 1-hexanol or 1-nonanol (10^2^ in mineraloil, all Sigma-Aldrich, St. Louis, USA). Two different odors were used as CS in order to avoid a potential odor identity bias. 100 μl of the diluted odor was applied to a cellulose stripe (SugiPad, Kettenbach GmbH KG, Eschenburg, Germany) located in a 3 ml syringe (Henke-Sass, Wolf GmbH, Tuttlingen, Germany). The odors were chosen based on previous studies^18^,^19^,^20^. Odor stimuli were delivered temporally precisely by utilizing a computer controlled olfactometer as described previously^32^. From two hours after training, bees were repeatedly fed to saturation with 1 M sugar water until the night before Ca^2+^-imaging or M17 recordings to ensure survival.

### Dnmt inhibitor treatment

2 hours after training 1 μl of the Dnmt inhibitor RG108 (Sigma-Aldrich, St. Louis, USA, 2 mM in DMF) or the solvent DMF was applied topically on the thorax as described previously^19^,^20^. RG108 treatment successfully reduces DNA methylation in the brain of honeybees^19^, Aplysia^27^ and mammals^28^,^29^,^30^. RG108 inhibits both Dnmt1 and Dnmt3 *in vitro* making it an unspecific Dnmt inhibitor^24^,^25^. In honeybees, a previous comparative study – using the same learning assay, stimuli, treatment method and time-point as used here - showed that two distinct Dnmt inhibitors (zebularine and RG108) reduce DNA methylation in the brain, affect the expression of memory-associated genes and impair stimulus-specific memory formation^19^. RG108 was chosen here as it was the more effective Dnmt inhibitor in that study. RG108 treatment does not affect stimulus perception (i.e. naïve odor or sugar responses), acquisition or short-term memory in bees arguing against unspecific effects of RG108^20^. Furthermore, unchanged responsiveness of bees to the CS+, odor mixture and control stimulus (mineral oil) in both M17 and Ca^2+^ measurements in this study suggests that bees’ general ability to respond to and process olfactory stimuli is not impaired by RG108 treatment. The treatment time-point was chosen based on previous studies for comparability^19^,^20^.

### Projection neuron staining and imaging

24 hours after training, the bees’ lateral and medial antenno-protocerebral tracts were stained with Fura-2 dextran (Invitrogen, ThermoFisher Scientific, Waltham, USA) - a Ca^2+^-sensitive dye - by inserting a dye crystal with a glass electrode between the mushroom body (MB) calyces, which are upstream of the PN dendrites and somata in the AL. Staining and preparation for imaging was done as described before^32^, with minor alterations. Although, staining with Fura-2 dextran is not expected to affect the ability of RG108 to block Dnmt activity and reduce DNA methylation levels, we chose to stain bees 24 hours after training in order to exclude this risk and any effect of staining on LTM formation. 24 hours after training (i.e. 22 hours after treatment) Dnmt-dependent changes in memory specificity, DNA methylation patterns and memory-associated gene expression are already established^18^,^19^,^20^. Additionally, Dnmts are up-regulated during the first hours after training, but at baseline levels 24 hours after^19^ suggesting that the sensitive period for learning-related DNA methylation is during the first hours after training, prior to the staining procedure performed here.

Calcium activity measured with the staining technique used here corresponds well with intracellular recordings of PNs and does not impair PN responses^45^. The brain was covered with bee saline solution (NaCl 130 mM, KCl 6 mM, MgCl_2_ 4 mM, CaCl_2_ 5 mM, Sucrose 160 mM D-Glucose 25 mM, HEPES 10 mM, pH 6.7). Bees were kept at room temperature overnight in the dark in a humid plastic container. Imaging of bees started 2 days after training. As 16–48 bees were trained each day the actual time between training and imaging for each individual bee differed. On average bees were imaged 52 hours after training (for more information see: Table 1). A total of 40 bees were imaged and analyzed (DMF paired: 9; RG108 paired: 13; DMF unpaired: 8; RG108 unpaired: 10). Supplemental Table 1 gives an overview of all bees discarded between treatment and measurement due to death (i.e. bees showed no movement and no response to mechanical and sugar stimulation), failure to record AL signals or technical issues (i.e. no staining, movement, and leakage). On average 79.7% of bees had to be excluded, because of death or lack of signal, which is indicative of dying of the animal or tissue. The rate was not different between RG108 and DMF treated bees (Fisher’s exact test, paired: p = 0.17, unpaired: p = 0.54). Although, the exclusion rate is high it is not unusual for this type of experiment, as bees were kept restrained for >48 hours and underwent stressful staining and dissection procedures.

Bees were imaged as described before^32^ with a spatial sampling rate of 172 × 130 pixel, using a 20× dip objective (NA = 0.95), and a Till-Imago CCD camera. Each recording lasted 16 s (200 frames) with the odor stimulus starting 4 s into the recording and lasting 4 s. Each frame was recorded with 340 and 380 nm excitation light at a rate of 12.5 Hz, thus one double frame lasted 80 ms. Odors were delivered during the measurement as described before^32^. Bees received an odor test first, consisting of the CS+, a new odor and mineraloil in randomized order followed by the binary mixture of CS+ and new odor (Fig. 1a). Odors were randomized during the test as stimuli order can affect the olfactory responses in the AL (e.g. due to adaptation to strong olfactory input). The odor test was followed by an extinction paradigm, consisting of a presentation of the CS+ 6 times and one presentation of mineral oil (solvent stimulus) at the end of the measurement as a contamination control (Fig. 1a). All stimuli were presented for 4 s (as during the training) and separated by 1 minute.

### M17 recordings

For M17 recordings, bees were stained as described above. 48 hours after training the M17 response was recorded. M17 activity correlates with the proboscis extension response (PER)^31^ and can therefore be used to assess memory retention. M17 activity is a more quantitative method of measuring learning in bees, as compared to visual observation of PER by an experimenter. One 0.2 mm insulated silver wire was inserted between the bee’s compound eye and lateral ocellus into the muscle, and a second one in the opposite eye as a reference (Fig. 1b). The signal was detected by a custom built digital oscilloscope with a resolution of 0.0625 ms, connected to the electrodes via an amplifier. Baseline spike frequency was measured for 5 s before every odor stimulus. Immediately afterwards spike activity during the 4 s odor stimulus was recorded. The spike frequency during the odor response was normalized with the frequency during each corresponding baseline measurement. The odor stimuli were the same as described above and shown in Fig. 1a. After the measurement, the bees’ PER was elicited by stimulating the antennae with 1 M sugar solution. All bees in which the M17 did not show activity in response to sugar were excluded. In sum, 73 bees were measured and analyzed (DMF paired: 26; RG108 paired: 19; DMF unpaired: 13; RG108 unpaired: 15). Supplemental Table 2 gives an overview of all bees discarded between the treatment and measurement due to death, no response to sugar or technical issues (e.g. electrode movement). On average 65.8% of bees had to be excluded, because of death or lack of sugar responses, which is indicative of dying or reduced health of the animal. The rate was not different between RG108 and DMF treated bees (Fisher’s exact test, paired: p = 0.28, unpaired: p = 0.44).

### Data analysis

All data analysis except the pre-processing of imaging data was done in R^46^. All scripts were custom written. M17 data was analyzed by extracting the number of spikes during the 5 s baseline and during the 4 s odor stimulus period. The M17 response frequency was calculated for each odor stimulus and was normalized with the corresponding baseline frequency.

Imaging data were pre-processed with the ImageBee plugin for KNIME^47^. Movement correction was performed for each bee first between images (i.e. frames) and then between videos (i.e. stimuli). Signals were calculated as the ratio of fluorescence at 340 and 380 nm: 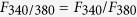. The *F*_*340/380*_ was then normalized to baseline levels by subtracting the average *F*_*340/380*_ of the first 40 frames (i.e. before odor onset). For glomeruli detection, videos were processed as follows: A Z-score normalization was performed, images were smoothed with a Gaussian filter, a principal component analysis was run and a convex cone algorithm was used as described elsewhere^47^. The map of glomeruli obtained by this procedure was than overlaid with the *F*_*340/380*_ calculations. The response of each glomerulus over time was calculated by averaging all pixels in the identified area. On average, 15 glomeruli could be analyzed per bee (Table 1). Bees which showed strong movement during one of the stimuli were excluded from the equivalent part of the analysis (i.e. test or extinction).

We calculated the Euclidean distance from the glomerular responses^48^ for each individual bee. We determined the glomeruli responding to each stimulus as described before^32^. All glomeruli exceeding 3× SD of the period before odor onset were counted as responsive. We determined the two most active glomeruli (dominant glomeruli) during the peak response for the CS+, new odor and first extinction trial for each individual bee. We pooled the response of those two glomeruli and calculated the mean and SEM across bees. We assessed the two strongest instead of all responding glomeruli as this method avoids introducing a bias caused by the reduced number of active glomeruli in the new odor after RG108 treatment (Fig. 3). Additionally, as each individual bee was trained with either 1-hexanol or 1-nonanol, the identity of the CS+ and new odor was different across bees. These two odors differ in which and how many glomeruli are activated^2^,^49^,^50^. Baseline response levels were not different between treatments or training groups (Supplemental Fig. 1).

We tested the data for normal distribution using Shapiro-Wilk tests and for equal variance using F-tests. Statistical significance was tested using a t-test, if data was normally distributed and had equal variance. Otherwise, data was tested using a non-parametric test: Mann-Whitney U for unpaired and Wilcoxon signed rank test for paired data. Two-tailed tests were used in all cases, except if a prior hypothesis about the directionality of an effect existed, in which case it is stated in the respective result section.

As bees were reared in their natural environment, inter-individual variation in olfactory responses is present and expected due to prior experiences of individual bees. Such variation allows us to identify biologically relevant effects of Dnmt activity in the bee brain. Importantly, this type of variation rather masks, than over-emphasizes effects on the group level.

The effect size (Cohen’s D) was calculated for all effects reaching the 0.05 significance level. As a guideline effects with sizes below 0.2 were defined as negligible, between 0.2–0.5 as small, between 0.5–0.8 as medium and above 0.8 as large^51^. The effect size can be used as an estimate of the real difference between the tested groups.

## Additional Information

**How to cite this article:** Biergans, S. D. *et al*. DNA methylation mediates neural processing after odor learning in the honeybee. *Sci. Rep.* **7**, 43635; doi: 10.1038/srep43635 (2017).

**Publisher's note:** Springer Nature remains neutral with regard to jurisdictional claims in published maps and institutional affiliations.

## Supplementary Material

Supplemental Material

## Figures and Tables

**Table 1:** Overview over number of bees (NoB), acquisition scores, measurement time, AL and glomeruli analysed.

	paired		unpaired	
	DMF	RG108	DMF	RG108
NoB (Ca2+)	9	13	8	10
NoB (M17)	26	19	13	15
Training PER (max. 6)	4.7 (+/−0.4)	4.3 (+/−0.5)	0.1 (+/−0.1)	0.2 (+/−0.2)
Time after training (h)	51.7 (+/−0.5)	51.7 (+/−0.8)	52.4 (+/−1.0)	51.3 (+/−0.8)
AL (1 = left; 0 = right)	0.4	0.6	0.9	0.5
Number of Glomeruli	14.2 (+/−1.0)	15 (+/−1.5)	15.5 (+/−2.5)	15 (+/−2.0)

For each group the mean (+/−SEM) across all bees used in the imaging experiment is shown for the accumulated CS+ response during 6 training trials, the time between t.
