# Supplementary material for: DNA methylation mediates neural processing after odor learning in the honeybee
Source: Sci Rep. 2017 Feb 27;7:43635. doi: 10.1038/srep43635 (PMC5378914; doi:10.1038/srep43635)
Supplement: Supplemental Material [file srep43635-s1.pdf]

DNA methylation mediates neural processing after odor learning in the honeybee

Stephanie D. Biergans[1,2*], Charles Claudianos[1,3], Judith Reinhard[1], C. Giovanni Galizia[2*]

[1] Queensland Brain Institute, The University of Queensland, Australia

[2] Neurobiologie, Universität Konstanz, Germany

[3] Monash Institute of Cognitive and Clinical Neuroscience, Faculty of Medicine, Nursing Health and Sciences, Monash University, Australia

Table S1 Bees excluded from final analysis of Ca^{2+} measurements due to: death during the 2 days between treatment and measurement, not showing AL signals or technical problems (i.e. no staining of the AL, strong movement or leakage of the preparation)

Treatment	Group	Dead (%)	No signal (%)	Technical issue (%)	In sum (%)
RG108	paired	56.4	20.5	12.0	88.9
	unpaired	57.3	24.0	5.3	86.7
DMF	paired	71.7	13.1	6.1	90.9
	unpaired	37.9	37.9	12.1	87.9

Table S2 Bees excluded from final analysis of M17 measurements due to: death during the 2 days between treatment and measurement, not showing response to sugar after measurement or technical problems (i.e. electrode moving or dislocating)

		Dead (%)	No sugar response (%)	Technical issue (%)	In sum (%)
RG108	paired	60.7	3.6	0	64.3
	unpaired	61.9	2.4	0	64.3
DMF	paired	46.3	13.4	1.5	59.7
	unpaired	73.1	1.9	0	75

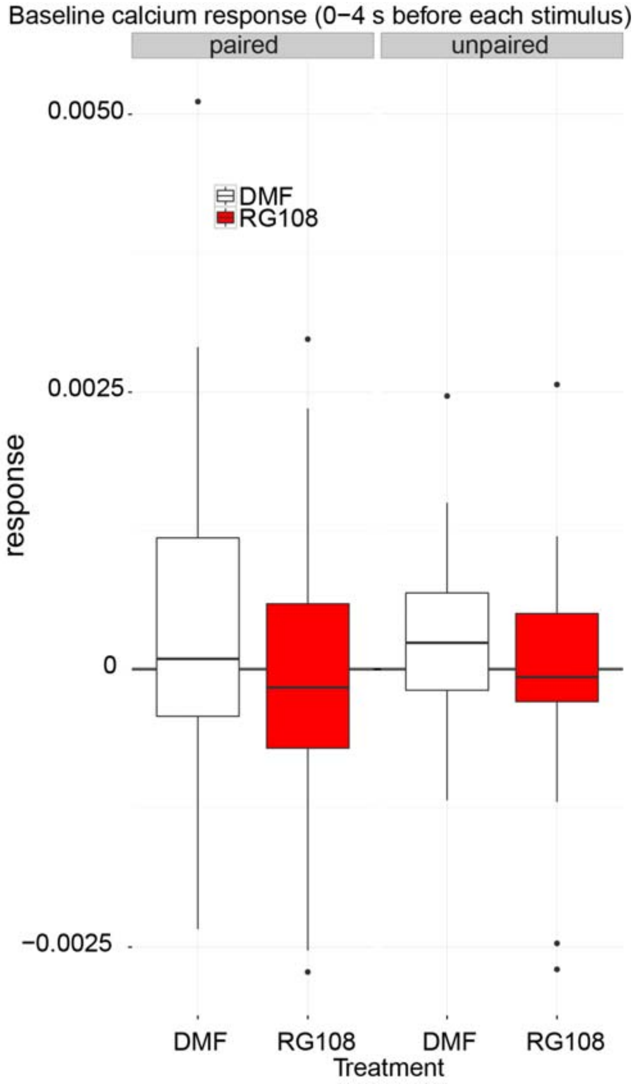

Figure S1 Baseline calcium responses measured during the first 4s before stimulus onset. Responses were pooled for each group and treatment across all measurements.
